# Circular Dichroism in Fluorescence Emission Following the C 1s→π* Excitation and Resonant Auger Decay of Carbon Monoxide

**DOI:** 10.3390/molecules23071534

**Published:** 2018-06-26

**Authors:** Martin Pitzer, Philipp Schmidt, Christian Ozga, Andreas Hans, Philipp Reiß, Ivan D. Petrov, Anton N. Artemyev, Arno Ehresmann, André Knie, Philipp V. Demekhin

**Affiliations:** 1 Institute of Physics and CINSaT, University of Kassel, Heinrich-Plett-Str. 40, 34132 Kassel, Germany; pitzer@atom.uni-frankfurt.de (M.P.); p.schmidt@uni-kassel.de (P.S.); ozga@physik.uni-kassel.de (C.O.); hans@physik.uni-kassel.de (A.H.); reiss@uni-kassel.de (P.R.); anton.artemyev@physik.uni-kassel.de (A.N.A.); ehresmann@physik.uni-kassel.de (A.E.); knie@physik.uni-kassel.de (A.K.); 2 Rostov State Transport University, Narodnogo Opolcheniya Square 2, 344038 Rostov-on-Don, Russia; petrov_id@mail.ru; 3 Research Institute of Physics, Southern Federal University, Stachki Avenue 194, 344090 Rostov-on-Don, Russia

**Keywords:** excitation of molecules, fluorescence spectra, polarization analysis, circular dichroism, partial wave analysis

## Abstract

Dichroism in angle-resolved spectra of circularly polarized fluorescence from freely-rotating CO molecules was studied experimentally and theoretically. For this purpose, carbon monoxide in the gas phase was exposed to circularly polarized soft X-ray synchrotron radiation. The photon energy was tuned across the C 1s→π* resonant excitation, which decayed via the participator Auger transition into the CO^+^ A ^2^Π state. The dichroic parameter β1 of the subsequent CO^+^ (A ^2^Π → X ^2^Σ^+^) visible fluorescence was measured by photon-induced fluorescence spectroscopy. Present experimental results are explained with the ab initio electronic structure and dynamics calculations performed by the single center method. Our results confirm the possibility to perform partial wave analysis of the emitted photoelectrons in closed-shell molecules.

## 1. Introduction

To a large extent, one-photon ionization of atoms and relatively small molecules by extreme ultraviolet and soft X-ray radiation can be described within the electric dipole approximation (see, e.g., Reference [1]). It assumes, that the incoming photon *γ* carries angular momentum of 1, and its projection on the propagation direction **k**_γ_ (known as helicity) is equal to 0 or ±1 for linear or circular (right- or left-handed) polarization, respectively. Thereby, simple angular momentum conservation rules for the emitted photoelectron and the electronic state of the remaining ion (known as selection rules) can be established. The transfer of the angular momentum and helicity of the ionizing photon to a target system results in two phenomena. On the one hand, it is responsible for the unique angular emission distribution of photoelectrons, which is an inherent property of a target. These angular distributions are given by coherent superpositions of the photoionization transition amplitudes for emission of the partial electron waves [2,3,4] that carry a particular angular momentum ℓ and its projection *m* on a chosen quantization axis. On the other hand, the emerging electronic state of an ion may become polarized, i.e., it can be aligned and oriented [2]. For atoms, the alignment 𝒜_20_ and orientation 𝒪_10_ parameters are defined with the help of the relative populations of the magnetic sublevels *M_L_* of the degenerate electronic states with angular momentum *L*. The aligned electronic state of an atom with 𝒜_20_ ≠ 0 possesses non-equal populations of the magnetic sub-levels with different |*M_L_*| values, yet equal for ±*M_L_*. In order to be oriented, i.e., 𝒪_10_ ≠ 0, the relative populations of magnetic sub-levels of an electronic state with ±*M_L_* should be different.

Information on those polarization parameters can be accessed via subsequent decay spectra of the remaining ion [2]. For instance, polarization parameters can be experimentally accessed by measuring spin-sensitive angle-resolved Auger electron spectra [5,6,7]. Alternatively, this can be done by a polarization analysis of angular distributions of subsequently emitted fluorescence photons [8,9,10,11,12]. For atoms and freely-rotating molecules in the gas phase ionized by circularly polarized radiation *γ*, the angular distribution of the subsequently emitted circularly polarized fluorescence *γ*′ is given by the following formula, Equation (1), for the differential fluorescence intensities [9,13].

(1) $$\frac{dI_{\pm \gamma^{\prime }}^{\pm \gamma }}{d\Omega }=\frac{1}{2}\frac{I}{4\pi }\left[1+\left(\pm 1\right)\left(\pm 1\right)\beta 1\;P_{1}\left(\theta \right)-\frac{1}{2}\beta 2\;P_{2}\left(\theta \right)\right],$$

where *θ* is the angle between the directions of the propagation of the ionizing **k**_γ_ and fluorescence **k**_γ′_ photons. Here, the two factors (±1) in front of the dichroic parameter β1 stand for the positive and negative helicities of the incoming ±γ and outgoing $\pm \gamma^{\prime }$ photons, respectively. In the most general case, the fluorescence dichroic (β1) and angular distribution (β2) parameters are given by coherent superpositions of the products of the photoionization and radiative decay transition matrix elements [9,13]. For a closed-shell atom, coherent sums can be analytically reduced to the incoherent linear combination of the partial photoionization cross sections [2,9], which determine relative populations of different magnetic sub-levels. Therefore, the ion polarization parameters become proportional to the dichroic and angular distribution parameters of fluorescence [2,9] as: 𝒪_10_ ~ β1 and 𝒜_20_ ~ β2. A similar result is obtained for the closed-shell diatomic molecules [13,14].

Angular emission distributions of fluorescence photons, as seen in Equation (1), emitted by an oriented electronic state of an ionized atom with 𝒪_10_ ≠ 0, exhibit a circular dichroism that is determined by β1. This circular dichroism in fluorescence spectra (FLCD) can only be observed if one is able to distinguish the right- and left-handed circularly polarized fluorescence photons $\pm \gamma^{\prime }$ for a particular emission angle by, e.g., a polarization analysis of the fluorescence. Moreover, the simultaneous measurement of the dichroic β1 and angular distribution β2 parameters of fluorescence enables the experimental determination of the relative photoionization cross sections for the emission of three partial electron waves, i.e., to perform a so-called partial wave analysis (PWA). PWA has already been performed experimentally for closed-shell atoms [10,11,12]. Note that performing PWA is not sufficient for a complete photoionization experiment [15], and an additional measurement of the photoelectron angular distribution is required to access relative phases of outgoing electron waves.

Recently [13], the possibility to perform PWA for the photoionization of closed-shell diatomic molecules by a simultaneous measurement of the β1 and β2 parameters for the subsequently emitted fluorescence has been proposed theoretically. The CO^+^ (A ^2^Π → X ^2^Σ^+^) fluorescence induced by photoionization of freely-rotating carbon monoxide was considered in the latter work as an illustrative example. In another work [14], this fluorescence had been investigated experimentally and theoretically in the soft X-ray ionization regime via the C 1s→π* resonant excitation and subsequent participator Auger decay into CO^+^ A ^2^Π (*v*′) vibronic states. Reference [14] made a first step towards the PWA of the photoionization process by measuring the respective angular distribution parameter β2 of the CO^+^ (A → X) fluorescence. In the present work, we report a joint experimental and theoretical study of circular dichroism in the CO^+^ (A → X) fluorescence spectra by measuring and computing the dichroic parameter β1. Thereby, we perform the final step towards a PWA of the C 1s→π* excitation and decay process of carbon monoxide. Taking into account that diatomic molecules cannot exhibit photoelectron circular dichroism, we conclude that here helicity of ionizing photons is fully transferred to the degenerate electronic states of the remaining ion and is reflected in its subsequent relaxation spectra.

## 2. Results and Discussion

We consider the following step-wise process, Equation (2), that leads to the emission of fluorescence [14]:

(2) $$\mathrm{CO}\;2\sigma^{2}1\pi^{4}5\sigma^{2}\left(X{\;}^{1}\Sigma^{+},\;v_{0}=0\right)+\hbar \omega \;↓\mathrm{Core}\;\mathrm{Excitation}\;C^{*}O\;2\sigma^{1}1\pi^{4}5\sigma^{2}2\pi^{1}{(}^{1}\Pi ,\;v_{r})\;↓\mathrm{participator}\;\mathrm{Auger}\;\mathrm{decay}/\mathrm{Direct}\;\mathrm{ionization}\;{\mathrm{CO}}^{+}\;2\sigma^{2}1\pi^{3}5\sigma^{2}\left(A{\;}^{2}\Pi ,\;v^{\prime }\right)+\varepsilon \ell m\;↓\mathrm{Fluorescence}\;\mathrm{emission}\;\mathrm{CO}\;2\sigma^{2}1\pi^{4}5\sigma^{2}\left(X{\;}^{1}\Sigma^{+},\;v^{\prime \prime }\right)+\varepsilon \ell m+hc/\lambda .$$

The synchrotron radiation with energy ℏω excits the CO molecule from its ground to core-excited vibronic states, which then undergo participator Auger decay into the CO^+^ A ^2^Π (*v*′) states. Thereby, electron partial waves εℓm are emitted. In addition to this resonant ionization pathway, the direct ionization process leading to the same final state of the ‘ion + free electron’ takes place (not explicitly shown in scheme (2) for brevity). The created vibronic states CO^+^ A ^2^Π (*v*′) decay further into the CO^+^ X ^2^Σ^+^ (*v*″) states via the emission of a fluorescence photon with wavelength *λ* in the visible range.

**Figure 1:**
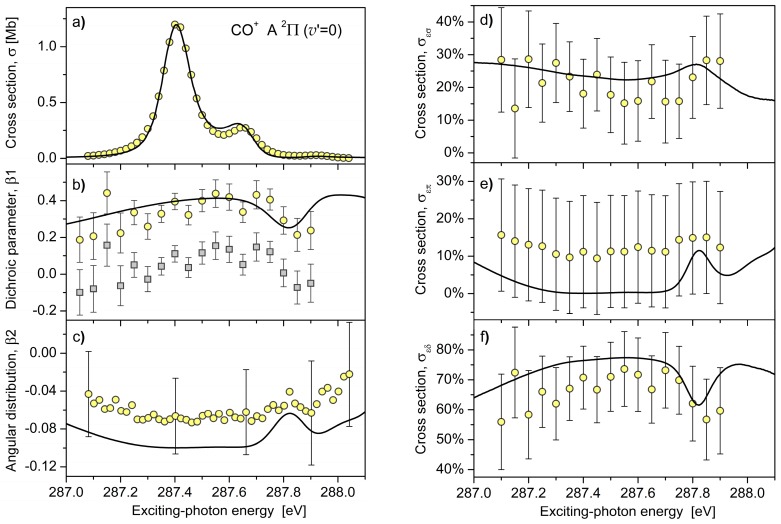
Comparison between the theory (solid curves) and experiment (open circles). Panel (**a**): cross section for the population of the CO^+^ A ^2^Π (*v*′ = 0) vibronic state in the vicinity of the C*O (1s→π*) resonance (data from Reference [14]). Panels (**b**,**c**): dichroic parameter β1 (present calculations and measurements) and angular distribution parameter β2 (data from Reference [14]) for the A ^2^Π (*v*′ = 0) → X ^2^Σ^+^ (*v*″ = 0) fluorescence band of the CO^+^ ion emitted after Auger decay of the C*O (1s→π*) resonance. The open squares in panel b) illustrate uncorrected data, while β1 values shown by open circles account for different transmissions of the circularly polarized fluorescence (see Section 3.3 for details). Panels (**d**–**f**): Relative partial photoionization cross sections (in percent from the total cross section shown in panel (**a**)) for the emission of three partial electron waves *ε*σ, *ε*π, and *ε*δ, resulting in the population of the CO^+^ A ^2^Π (*v*′ = 0) vibronic state (obtained via Equation (3) by combining the present data for β1 and data from Reference [14] for β2).

Dichroic parameters β1, computed and measured in the present work for the A ^2^Π (*v*′ = 0) → X ^2^Σ^+^ (*v*″ = 0) fluorescence band (*λ*_00_ ≈ 490 nm [16]), emitted in the last step of the cascade decay (2), are depicted in Figure 1b as functions of the exciting-photon energy. For a better overview, Figure 1a,c depict the cross sections for the population of the initial fluorescence state and the angular distribution parameters β2 of this fluorescence, which were computed and measured in our previous work [14]. Figure 1b illustrates that the trend between the β1(*ω*) dispersions, computed and measured across the *v_r_* = 0, 1, and 2 vibrational levels of the C 1s→π* resonance, agree very well. The theory predicts about 40% asymmetry in the forward/backward emission of the circularly polarized fluorescence photons after the C 1s→π* excitation of carbon monoxide by circularly polarized synchrotron radiation. However, the measured values of β1(ω) are systematically lower than the computed ones (cf., solid line and open squares in Figure 1b). This deviation can be explained by accounting for different transmissions of the circularly polarized fluorescence through the spectro-polarimeter (see Section 3.3 for details). As a consequence, the presently computed and measured dichroic parameters agree within the experimental uncertainties (cf., solid line and open circles in Figure 1b). Depolarization of the initial electronic state of fluorescence during the respective radiative decay lifetime (estimated for the present case to be a few μs [14]) can be responsible for a systematic offset between the present theoretical and experimental data. Those disalignment and disorientation are usually caused by: (i) additional population of the initial state via radiative cascades of highly-excited ionic states produced by the Auger decay [9]; and (ii) coupling between the electronic and nuclear rotational degrees of freedom, which induces precession of the electronic angular momentum around the resulting total momentum [17].

As discussed in the introduction, simultaneous measurement of parameters β1 and β2 allows for a determination of the relative cross sections for the emission of three partial electron waves (to perform a PWA). The initial state of fluorescence CO^+^ A ^2^Π can be populated via the photoionization of the 1π-electron into the εσ-, επ-, and εδ-waves. For the subsequent ^2^Π → ^2^Σ radiative decay, the respective relative cross sections are related to β1 and β2 parameters via [13]:

(3) $$\begin{array}{ll} \sigma_{\varepsilon \sigma }=\frac{1}{3}-\frac{2}{3}\beta 1-\frac{5}{3}\beta 2, & \;\left(a\right)\\ \sigma_{\varepsilon \pi }=\frac{1}{3}\;+\frac{10}{3}\beta 2, & \;\left(b\right)\\ \sigma_{\varepsilon \delta }=\frac{1}{3}+\frac{2}{3}\beta 1-\frac{5}{3}\beta 2, & \;\left(c\right) \end{array}$$

Here, all cross sections *σ*_ελ_ are normalized to their sum at each exciting-photon energy, such that the sum of the relative cross sections yields unity. Figure 1d–f depict these relative cross sections (in per cent), extracted via Equation (3) from the experimental data shown in Figure 1b,c. This PWA was possible only at the exciting-photon energies where both parameters, β1 from the present experiment and β2 from the experiment performed in our previous work [14], were available. Note also that β1 values corrected for different transmissions of the circularly polarized fluorescence (open circles) were used in the present PWA. One can see from Figure 1d–f that the experimental cross sections *σ*_ελ_ agree (within the error bars) with the presently computed theoretical cross sections. In the considered energy range, the emission of the εδ-wave dominates over the emission of εσ- and επ-waves. The επ-wave has the smallest emission probability, and it is hardly emitted in the on-resonance excitation regime. This result can be rationalized as follows. Here, the resonant ionization pathway via excitation and decay of the intermediate resonance dominates over the direct ionization pathway. The strong resonant channel generates only εσ- and εδ-waves (the total symmetry of the ‘ion + electron’ should be equal to the symmetry ^1^Π of the resonance), while the *ε*π-wave can only be emitted via the weak direct channel. The present experimental results in Figure 1d–f confirm the latter theoretical justification.

## 3. Materials and Methods

### 3.1. Theory

The calculations of the angle-resolved fluorescence spectra emitted during the cascade decay (2) were based on the theoretical approach reported in our previous work [14]. Briefly, the electronic transition amplitudes for the excitation, Auger decay, direct ionization, and radiative decay of carbon monoxide were computed by the single center (SC) method and code [18,19,20], which enables an accurate theoretical description of the molecular photoionization processes. Calculations were performed at the equilibrium internuclear distance of the ground electronic state of CO within the relaxed-core Hartree-Fock approximation, including monopole relaxation of molecular orbitals. The one dimensional nuclear dynamics calculations were performed with the help of the relevant potential energy curves reported in Figure 1 of Reference [14]. In the calculations, electronic-state interference (ESI, [21]) between the resonant and direct ionization pathways, as well as lifetime vibrational interference (LVI, [22]) of the excited vibrational levels overlapping within their natural decay widths, were taken into account via Equation (18) of Reference [14]. For the CO^+^ A ^2^Π (*v*′ = 0) vibronic state, the impact of ESI and LVI on the angular emission distributions of photoelectrons and fluorescence photons is illustrated in Figure 1 of Reference [14]. Fluorescence dichroic parameter β1 was computed via Equation (3a) of Reference [13]. More details on the present theory can be found in References [13,14,18].

### 3.2. Experiment

To measure the degree of circular polarization of fluorescence photons, we expanded our typical photon-induced fluorescence spectrometer set-up [10,12,14,23,24,25,26] with a newly-developed spectro-polarimeter. In the original set-up, dispersive 1m-normal-incidence-spectrometers were combined with position sensitive detectors to resolve the emission spectra of photoexcited atoms and molecules [23,24,25,26]. This set-up can easily be enriched to measure the degree of linear polarization of the emitted photons via a Wollaston prism displacing the two linear polarization components of the transmitted light [14]. Such measurements can be performed in the dipole plane, i.e., perpendicular to the direction of the propagation of the exciting radiation **k**_γ_. Here, as we intended to measure the degree of circular polarization, the detection direction **k**_γ′_ needed to be out of the dipole plane, optimally close to **k**_γ_. At the same time, the emission angle needed to be close to 54.73° to reduce the impact of the angular distribution parameter β2. As a practical compromise, we designed the interaction chamber and gas cell in such a way that the emitted fluorescence was collected in a forward direction at an angle of $\theta =45^{{}^{\circ}}$ with respect to the direction of the propagation of exciting radiation.

**Figure 2:**
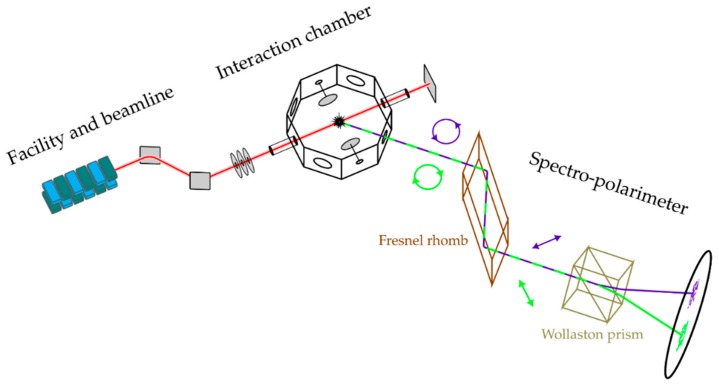
An overview of the experimental set-up illustrating the experimental geometry, design of the interaction chamber and spectro-polarimeter with details of the polarization analysis (see text).

A sketch of the present set-up is depicted in Figure 2. The tunable elliptically polarized synchrotron radiation from the UE56/2 PGM2 beamline at BESSY II (Helmholtz Zentrum Berlin) was transmitted via a differential pumping stage into the interaction chamber. The light was focused into the gas cell where it excited the CO molecules. The target pressure was regulated by an electronically steered valve and a capacitance Baratron gauge from MKS (Andover MA, USA) to a value of 25 Pa. Simultaneously, the photocurrent and the transmitted synchrotron radiation were measured by the insulated mounted electrodes and a Faraday cup (or a photodiode in the beam dump), respectively. Emitted fluorescence photons escaped the gas cell through a collimating lens (f = 35 mm). In order to make the set-up more versatile and to be able to investigate a wide range of different wavelengths, a Fresnel rhomb was used here instead of a quarter-wave plate. The Fresnel rhomb converted the two circularly polarized components into two mutually-orthogonal linearly polarized components. The linear polarizations were then spatially separated by a Wollaston prism with an opening angle of 10°. Since this geometry is difficult to combine with a 1m-normal-incidence spectrometer, a transmission grating or wavelength filters can be used downstream of the Wollaston prism. Here, a Balmer beta filter from Astronomik (Hamburg, Germany), which transmits only photons within a wavelength range of 486–505 nm, was used. The beam was then focused onto the position sensitive detector.

### 3.3. Data Analysis

The present measurements were carried out for the A ^2^Π (*v*′ = 0) → X ^2^Σ^+^ (*v*″ = 0) fluorescence band emitted at around 490 nm [16]. This visible fluorescence was excited by the right-handed circularly polarized synchrotron radiation (+γ) with the average Stokes parameter $S_{3}\approx 0.45$ [27]. With the present experimental geometry ($\theta =45^{{}^{\circ}}$), the degree of circular polarization of the fluorescence photons ($\pm \gamma^{\prime }$) is given by the following formulae (see Equation (2.17b) in Reference [2] and Equation (1) here):

(4) $$P_{\mathrm{circ}}=\frac{I_{+\gamma^{\prime }}^{+\gamma }-I_{-\gamma^{\prime }}^{+\gamma }}{I_{+\gamma^{\prime }}^{+\gamma }+I_{-\gamma^{\prime }}^{+\gamma }}=\frac{S_{3}\beta 1P_{1}\left(\theta \right)}{1-\frac{1}{2}\beta 2P_{2}\left(\theta \right)}=\frac{S_{3}\beta 1}{\sqrt{2}\left(1-\beta 2/8\right)}≅\frac{S_{3}\beta 1}{\sqrt{2}}.$$

The approximation made at the end of Equation (4) is well justified. Indeed, in the considered exciting-photon energy range, the respective angular distribution parameter β2 ranges in between −0.05 and −0.08 [14] (see also Figure 1d). Therefore, as a very good approximation, the factor (1−β2/8) can be set to 1. Before extracting the dichroic parameter via Equation (4), the recorded fluorescence intensities $I_{\pm \gamma^{\prime }}^{+\gamma }$ were corrected for the background fluorescence present in both spectra due to the linearly polarized and unpolarized beam contaminations, as well as the signal induced by secondary scattering processes. The experimental uncertainties were obtained by error propagation from the statistical number of photon counts and take into account corrections for those contaminations.

The transmission efficiencies for the left- and right-handed circularly polarized fluorescences depend on the rotation angle of the spectro-polarimeter around the fluorescence propagation direction. The correct orientation was checked prior to the measurement by using a linearly polarized exciting beam that resulted in equal fractions of the left- and right-handed circularly polarized fluorescence. However, the transmission of left- and right-handed circularly polarized fluorescence through the spectro-polarimeter is equal for photons emitted on the optical axis only. Here, an important issue of the spatial displacement of the beamline focus arises when exchanging linear polarization of the exciting synchrotron radiation with the circular polarization. Such a displacement of the beamline focus (and, as a consequence, of the region from where fluorescence is emitted) cannot be ruled out through measurement with linear polarization and was therefore difficult to avoid. In the detector image, a shift of the spots by about 1 mm was visible when exchanging polarization of the exciting synchrotron radiation. It amounts to about 20% of a total spot size of around 5 mm. This value can be considered as an estimate for the difference in the transmissions of the left- and right-handed circularly polarized fluorescence through the spectro-polarimeter. Figure 1b illustrates that taking into account 20% difference in the transmissions leads to an excellent agreement between the experimental and theoretical results (cf., open circles and solid lines).

## 4. Conclusions and Outlook

It was demonstrated that angle-resolved emission spectra of circularly polarized fluorescence, emitted by freely-rotating diatomic molecules ionized by circularly polarized radiation, exhibit sizable dichroism (FLCD). In particular, the C 1s→π* excitation and subsequent cascade decay that resulted in the emission of the CO^+^ (A → X) fluorescence was considered experimentally and theoretically. The measured dichroic parameter β1(ω), together with the angular distribution parameter β2(ω) measured in our previous work [14], enabled us to perform partial wave analysis for the C 1s→π* excitation and resonant Auger decay of carbon monoxide. These experimental results agree well with the outcome of the present theoretical calculations.

It is well-known [28], that angular emission distributions of photoelectrons emitted by freely-rotating linear molecules do not exhibit circular dichroism (PECD). Here, because of the molecular symmetry, a complete compensation of the contributions from the partial electron waves with different projections ±*m* takes place for the dichroic parameter of photoelectrons [28]. The results presented here allow us to conclude that, in this case, circular dichroism of the ionizing photon is fully transferred to the remaining ion, whose degenerate electronic states with well-defined magnetic quantum number *M_L_* become oriented (polarized). As a consequence, circular dichroism cannot be observed in the photoelectron spectra, but rather in the spectra for subsequent relaxation of the ion, e.g., as the FLCD.

On the contrary, non-degenerate electronic states of chiral molecules without any symmetry (C1-symmetry) cannot be oriented by circularly polarized radiation. As a result, spectra for subsequent relaxation of the chiral ion cannot possess circular dichroism (e.g., FLCD). Here, circular dichroism of the ionizing photon is fully transferred to the photoelectron [28]. However, chiral molecules with rotational symmetries may have degenerate electronic states (e.g., 3d-orbitals of the transition-metal center in propeller-shaped (helical) metal-organic complexes with D_3_-symmetry). Here, an interesting question of whether the circular dichroism of the ionizing photon can be shared between the photoelectron and remaining ion, and both PECD and FLCD phenomena can be observed, remains open for further investigations.

## References

[1] Bethe H.A., Salpeter E.E. (1957). Quantum Mechanics of One- and Two-Electron Atoms.

[2] Schmidt V. (1992). Photoionization of atoms using synchrotron radiation. Rep. Prog. Phys..

[3] Cherepkov N.A. (1981). Theory of spin polarisation phenomena in molecular photoionisation processes. J. Phys. B.

[4] Cherepkov N.A. (1982). Circular dichroism of molecules in the continuous absorption region. Chem. Phys. Lett..

[5] Snell G., Langer B., Drescher M., Müller N., Zimmermann B., Hergenhahn U., Viefhaus J., Heinzmann U., Becker U. (1999). Complete Description of the Xe 4d Photoionization by Spin-Resolved Photoelectron and Auger Spectroscopy. Phys. Rev. Lett..

[6] Schmidtke B., Khalil T., Drescher M., Müller N., Kabachnik N.M., Heinzmann U. (2000). Testing the feasibility of a complete Auger decay experiment by spin- and angle-resolved electron spectroscopy on Xe N_4_O_2,3_O_2,3_
^3^P_1_. J. Phys. B.

[7] Schmidtke B., Khalil T., Drescher M., Müller N., Kabachnik N.M., Heinzmann U. (2001). The Kr M_4,5_N_1_N_2,3_
^1^P_1_ Auger decay: Measurement of the transferred spin polarization and analysis of Auger amplitudes. J. Phys. B.

[8] Lagutin B.M., Petrov I.D., Sukhorukov V.L., Kammer S., Mickat S., Schill R., Schartner K.-H., Ehresmann A., Shutov Y.A., Schmoranzer H. (2003). Raman regime energy dependence of alignment and orientation of kr ii states populated by resonant auger effect. Phys. Rev. Lett..

[9] Lagutin B.M., Petrov I.D., Sukhorukov V.L., Demekhin P.V., Zimmermann B., Mickat S., Kammer S., Schartner K.-H., Ehresmann A., Shutov Y.A. (2003). The interference effects in the alignment and orientation of the Kr II 4p^4^5p states following Kr I 3d^9^np resonance excitation. J. Phys. B.

[10] Schill R., Hasselkamp D., Kammer S., Mickat S., Zimmermann B., Schartner K.-H., Ehresmann A., Schmoranzer H., Schlüter M., Shutov Y.A. (2003). Partial wave analysis of the Kr I 3d^9^_5/2_5p_3/2_→Kr II 4p^4^(^1^D)5p ^2^F_7/2_ decay, based on orientation and alignment transfer. J. Phys. B.

[11] Schartner K.-H., Schill R., Hasselkamp D., Mickat S., Kammer S., Werner L., Klumpp S., Ehresmann A., Schmoranzer H., Lagutin B.M. (2005). Partial wave analysis of interfering Kr 3d^9^5p resonant Raman Auger transitions based on measurements of alignment and orientation parameters within the natural line width. J. Phys. B.

[12] Schartner K.-H., Schill R., Hasselkamp D., Mickat S., Kammer S., Werner L., Klumpp S., Ehresmann A., Schmoranzer H., Lagutin B.M. (2007). Interference between resonant Raman Auger decay and direct excitation manifested in orientation and alignment of KrII 4p^4^(^1^D)5p ^2^P_3/2_ ions. J. Phys. B.

[13] Demekhin P.V., Petrov I.D., Ehresmann A. (2010). Partial-photoelectron-wave analysis in diatomic molecule photoionization by fluorescence polarization experiments. Phys. Rev. A.

[14] Demekhin P.V., Petrov I.D., Sukhorukov V.L., Kielich W., Reiß P., Hentges R., Haar I., Schmoranzer H., Ehresmann A. (2009). Interference effects during the Auger decay of the C*O(1s−1π*) resonance studied by angular distribution of the CO^+^(A) photoelectrons and polarization analysis of the CO^+^(A→X) fluorescence. Phys. Rev. A.

[15] Cherepkov N.A., Semenov S.K. (2004). On a complete experiment on photoionization of atoms. J. Phys. B.

[16] Krupenie P.H. (1966). The band spectrum of carbon monoxide. Natl. Stand. Ref. Data Ser..

[17] Blum K. (1996). Density Matrix Theory and Applications.

[18] Demekhin P.V., Ehresmann A., Sukhorukov V.L. (2011). Single center method: A computational tool for ionization and electronic excitation studies of molecules. J. Chem. Phys..

[19] Demekhin P.V., Omel’yanenko D.V., Lagutin B.M., Sukhorukov V.L., Werner L., Ehresmann A., Schartner K.-H., Schmoranzer H. (2007). Investigation of photoionization and photodissociation of an oxygen molecule by the method of coupled differential equations. Opt. Spektrosc..

[20] Galitskiy S.A., Artemyev A.N., Jänkälä K., Lagutin B.M., Demekhin P.V. (2015). Hartree-Fock calculation of the differential photoionization cross sections of small Li clusters. J. Chem. Phys..

[21] Cesar A., Ågren H. (1992). State interference in resonance Auger and x-ray emission. Phys. Rev. A.

[22] Gel’mukhanov F.K., Mazalov L.N., Kondratenko A.V. (1977). A theory of vibrational structure in the X-ray spectra of molecules. Chem. Phys. Lett..

[23] Schmoranzer H., Liebel H., Vollweiler F., Müller-Albrecht R., Ehresmann A., Schartner K.-H., Zimmermann B. (2001). Photon-induced fluorescence spectroscopy (PIFS). Nucl. Instrum. Methods Phys. Res. A.

[24] Ozga C., Reiss P., Kielich W., Klumpp S., Knie A., Ehresmann A. (2015). Fluorescence cascades after excitation of XeII 5p^4^6p satellite states by synchrotron radiation. J. Phys. B.

[25] Hans A., Knie A., Schmidt P., Ben Ltaief L., Ozga C., Reiß P., Huckfeldt H., Förstel M., Hergenhahn U., Ehresmann A. (2015). Lyman-series emission after valence and core excitation of water vapor. Phys. Rev. A.

[26] Hans A., Schmidt P., Ozga C., Hartmann G., Holzapfel X., Ehresmann A., Knie A. (2018). Extreme ultraviolet to visible dispersed single photon detection for highly sensitive sensing of fundamental processes in diverse samples. Materials.

[27] Gaupp A., Schäfers F., MacDonald M., Uschakow S., Salashchenko N.N., Gaykovich P.K. (2013). Carbon K-edge polarimetry with Cr/Sc multilayers. J. Phys. Conf. Ser..

[28] Ritchie B. (1976). Theory of the angular distribution of photoelectrons ejected from optically active molecules and molecular negative ions. Phys. Rev. A.

